# Earthquake Damage Visualization (EDV) Technique for the Rapid Detection of Earthquake-Induced Damages Using SAR Data

**DOI:** 10.3390/s17020235

**Published:** 2017-01-27

**Authors:** Ram C. Sharma, Ryutaro Tateishi, Keitarou Hara, Hoan Thanh Nguyen, Saeid Gharechelou, Luong Viet Nguyen

**Affiliations:** 1 Center for Environmental Remote Sensing (CEReS), Chiba University, 1-33 Yayoi-cho, Inage-ku, Chiba 263-8522, Japan; tateishi@faculty.chiba-u.jp (R.T.); acfa3286@chiba-u.jp (S.G.); 2 Department of Informatics, Tokyo University of Information Sciences, 4-1 Onaridai, Wakaba-ku, Chiba 265-8501, Japan; hara@rsch.tuis.ac.jp; 3 Institute of Geography, Vietnam Academy of Science and Technology, 18 Hoang Quoc Viet Str., Cau Giay Dist., Hanoi 10000, Vietnam; hoanrs@gmail.com; 4 Department of Civil Engineering, Shahrood University of Technology, Shahrood 3619995161, Iran; sgharachelo@gmail.com; 5 Space Technology Institute, Vietnam Academy of Science and Technology, 18 Hoang Quoc Viet Str., Cau Giay Dist., Hanoi 10000, Vietnam; nvluong@sti.vast.vn

**Keywords:** earthquake damage, coherence, visualization, EDV, 2015 Nepal Earthquake, ALOS-2, SAR, cross-validation, buildings

## Abstract

The damage of buildings and manmade structures, where most of human activities occur, is the major cause of casualties of from earthquakes. In this paper, an improved technique, Earthquake Damage Visualization (EDV) is presented for the rapid detection of earthquake damage using the Synthetic Aperture Radar (SAR) data. The EDV is based on the pre-seismic and co-seismic coherence change method. The normalized difference between the pre-seismic and co-seismic coherences, and vice versa, are used to calculate the forward (from pre-seismic to co-seismic) and backward (from co-seismic to pre-seismic) change parameters, respectively. The backward change parameter is added to visualize the retrospective changes caused by factors other than the earthquake. The third change-free parameter uses the average values of the pre-seismic and co-seismic coherence maps. These three change parameters were ultimately merged into the EDV as an RGB (Red, Green, and Blue) composite imagery. The EDV could visualize the earthquake damage efficiently using Horizontal transmit and Horizontal receive (HH), and Horizontal transmit and Vertical receive (HV) polarizations data from the Advanced Land Observing Satellite-2 (ALOS-2). Its performance was evaluated in the Kathmandu Valley, which was hit severely by the 2015 Nepal Earthquake. The cross-validation results showed that the EDV is more sensitive to the damaged buildings than the existing method. The EDV could be used for building damage detection in other earthquakes as well.

## 1. Introduction

Earthquakes are one of the most catastrophic natural disasters. More than 23 million deaths have been caused by earthquakes during the period of 1902–2011 alone globally (http://earthquake.usgs.gov, last accessed 15 September 2016). Earthquake-resistant buildings and infrastructures, and emergency services during disasters are vital for protecting against earthquake damage. However, mass deaths and injuries caused by earthquakes over the past decades undermine the present situation. While urbanization is inevitable as a cost of the rising population, damage reduction strategies, particularly in vulnerable fault zones, are mandatory. Satellite remote sensing is an important technology for the monitoring of disasters.

Buildings’ damage information is essential for rescue, humanitarian and reconstruction operations in the disaster area [1]. The space-borne optical and Synthetic Aperture Radar (SAR) data have been used for building damage assessment in a number of places—for example, the 1995 earthquake in Kobe, Japan [2], the 1999 earthquake in Izmit, Turkey [3], the 2001 earthquake in Gugrat, India [4,5], the 2003 earthquake in Bam, Iran [6,7,8,9], the 2005 earthquake in Azad Kasmir, Pakistan [10], the 2006 earthquake in Java, Indonesia [11], the 2008 earthquake in Sichuan, China [12,13,14,15], the 2009 earthquake in L’Aquila, Italy [16], and the 2010 earthquake in Haiti [17,18,19]. Building damage has been ranked in the field using damage scales [20,21,22]; however, remotely sensed images are mostly capable of detecting heavy damage levels only, i.e., totally collapsed buildings [23,24].

The near real-time disaster mapping using satellite data is important for minimizing the effects of natural disasters [25]. Very-high-resolution optical imagery can visualize earthquake damage, and it has been used by many researchers [9,12,26,27]. Major techniques used for retrieving the earthquake-induced building damage information are object based classification [28,29,30], template matching and pattern recognition [31], and supervised classification [32,33,34]. Airborne imagery is another source of building damage information [35,36,37,38,39,40]. In some research, a combination of SAR and optical data has provided better results [14,41]. However, availability of the optical images when urgently needed can not be guaranteed due to clouds and atmospheric conditions. SAR data with negligible atmospheric effects are considered more appropriate for the detection and mapping of the earthquake damage. SAR backscattering intensity data have shown some successes in detecting damage [42,43,44]. More recently, Yun et al. [45] have produced the NASA Damage Proxy Map (NDPM) based on pre-seismic and co-seismic coherence change methods using SAR data.

This paper presents an improved technique called the Earthquake Damage Visualization (EDV) for rapid detection of earthquake induced damage. The EDV technique builds on the existing NDPM method by using additional polarization and more change parameters. The performance of the EDV technique is demonstrated in the case of the 2015 Nepal Earthquake using the ALOS-2 SAR data and compared it to the existing NDPM method.

## 2. Methodology

### 2.1. Study Area

**Figure 1:**
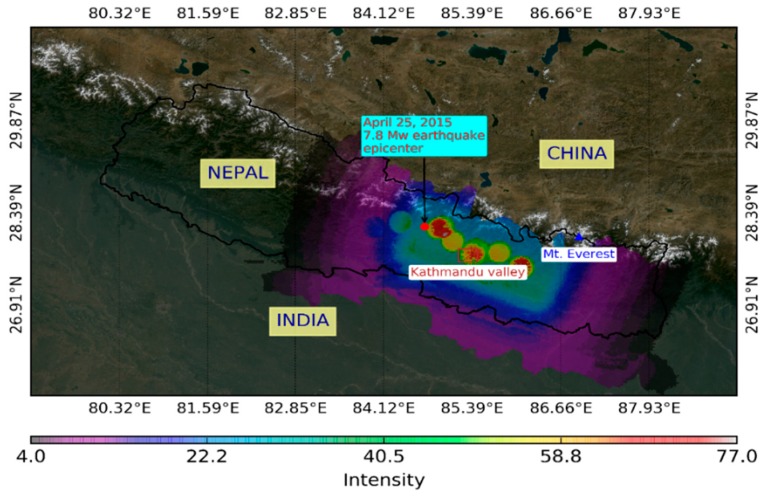
Location map of the study area, the Kathmandu Valley (red polygon) displayed with the shake areas.

This research was carried out in the urban areas of the Kathmandu Valley, which was severely hit by a 7.8 Mw earthquake on 25 April 2015. The Kathmandu Valley is a bowl-shaped valley standing at 1425 m above sea level, and surrounded by mountainous ranges. The location map of the study area is displayed in Figure 1. The major shake areas based on Mercalli intensity data of the earthquake (http://earthquake.usgs.gov, last accessed 15 September 2016) are also plotted in Figure 1, which shows that the Kathmandu Valley was shaken severely by the earthquake.

### 2.2. Proposal of the EDV

The EDV is based on estimates of the coherence changes between pre-seismic and co-seismic timings. A pair of before earthquake (pre-seismic) images are used to calculate the pre-seismic coherence; whereas a pair of before and after earthquake (co-seismic) images are used to calculate the co-seismic coherence. A coherence map shows similarity (cross-correlation) between two radar images pixel by pixel. High coherence values means that the ground objects remained the same between the two timings of the radar image acquisition, whereas low coherence means that the objects changed during that time. The pre-seismic and co-seismic coherence maps are then used to calculate forward (from pre-seismic to co-seismic) change, backward (from co-seismic to pre-seismic) change, and no change (between pre-seismic and co-seismic) parameters. The normalized difference between the pre-seismic and co-seismic coherences, and vice versa, are calculated to derive the forward and backward change parameters, respectively. The backward change parameter is added to see the retrospective changes caused by factors other than the earthquake. The average values of the pre-seismic and co-seismic coherences are calculated as the third change-free parameter. These three change parameters are ultimately merged as the RGB (Red Green and Blue) color composite imagery, called EDV.

**Figure 2:**
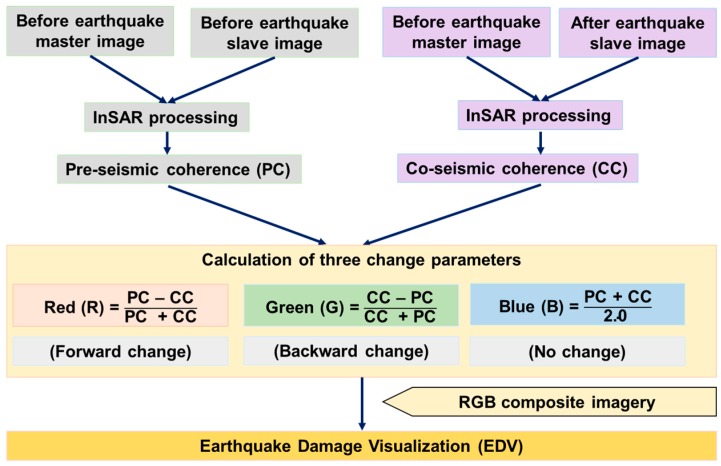
Flow chart showing creation of the Earthquake Damage Visualization (EDV).

Advanced Land Observing Satellite-2 (ALOS-2) based L-band Single Look Complex (SLC) SAR images available from the Japan Aerospace Exploration Agency (JAXA) were used in the research. A pair of pre-seismic (4 October 2014 and 21 February 2015) images and another pair of co-seismic (21 February 2015 and 2 May 2015) images were used to calculate pre-seismic and co-seismic coherences, respectively. The pre-seismic and co-seismic coherences were calculated separately for the Horizontal transmit and Horizontal receive (HH), and Horizontal transmit and Vertical receive (HV) polarizations and averaged together for creating the EDV. The procedure of calculating the EDV for a single polarization is described in Figure 2.

### 2.3. Validation Approach

The performance of the EDV was evaluated by using the k-fold cross-validation method. The k-fold method divides all the samples into k groups of samples, called folds. The k-1 folds are used for learning, whereas the remaining one fold is used for the validation. The total number of folds used in this validation was 10, so it is called the 10- fold cross-validation method. Four commonly used supervised classifiers (k-Nearest Neighbors, Gaussian Naive Bayes, Random Forests, and Support Vector) were tested for the cross-validation. Readers are referred to the OpenCV (http://opencv.org)—an optimized C/C++ programming library for computer vision, machine learning and robotics—for in-depth description of the supervised classifiers used.

**Figure 3:**
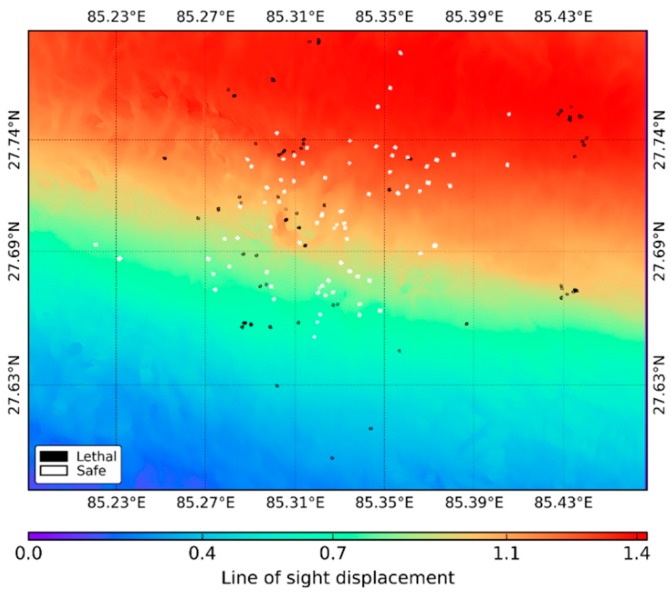
Distribution of the ground truth polygons in the Kathmandu Valley displayed over the line of sight displacement image resulted from differential interferometric processing in the research.

For the cross-validation, ground truth data were prepared considering the European Macroseismic Scale 1998 (EMS-1998, [46]). The EMS-1998 categorizes strength of an earthquake that affects a specific location, and it has 12 divisions. However, from the viewpoint of casualties and injuries caused by earthquake induced damage, and considering the capability of currently available satellite based SAR data, we generalized two classes in the research: safe and lethal. Divisions 1 (Not felt) to 7 (Damaging) were grouped as a safe class; whereas divisions 8 (Heavily damaging) to 12 (completely devastating) were grouped as the lethal class. Ground truth polygons belonging to lethal and safe blocks of buildings, historical monuments, towers, and temples were prepared through visual inspection. Each class consists of 90 polygons. The distribution of the ground truth polygons is shown in Figure 3 over the line of sight displacement map resulting from the differential interferometric processing carried out in the research. The displacement map shows that the ground surface uplifted up to 1.4 m in the northern part of the Kathmandu Valley due to the earthquake (Figure 3).

The performance of the EDV was compared to the NASA Damage Proxy Map (NDPM) using the ground truth polygons. The NDPM map is available online for downloading from http://aria-share.jpl.nasa.gov/events/20150425-Nepal_EQ/DPM/ (last accessed 15 September 2016). Cross-validation of the EDV and NDPM methods were carried out based on 90 ground truth polygons available for each class (lethal or safe). The ground truth polygons belonging to two classes (lethal or safe) were laid over the raster images (EDV or NDPM), and the pixels delineated by a polygon were extracted and their median values were calculated. The number of pixels falling inside a polygon varies according to the size of building block. The median pixel data calculated for each layer of the EDV and NDPM images were used for the cross-validation. The EDV has three layers, whereas the NDPM has a single layer. Google Earth based time-lapse images were used to visualize performance of the EDV and NDPM images.

## 3. Results and Discussion

### 3.1. Performance of the EDV

**Figure 4:**
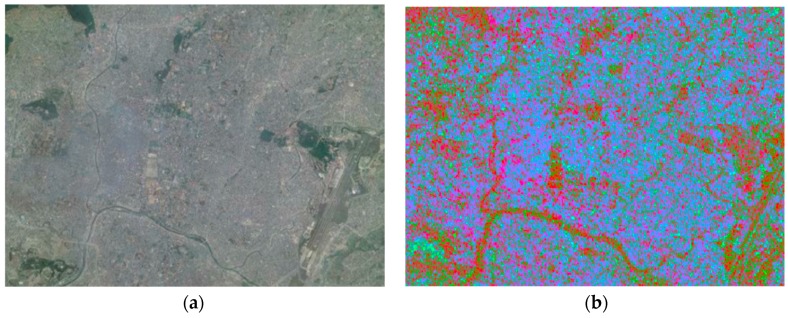
Earthquake Damage Visualization (EDV) imagery showing forward (red), backward (green), and change-free (blue) components: (**a**) Google map imagery of the Kathmandu Valley dated 3 May 2015; (**b**) the corresponding EDV imagery.

The EDV created from the ALOS-2 SAR data over the Kathmandu Valley is displayed in Figure 4. The EDV has three components: red, green, and blue. The coherence changes in the forward direction (from pre-seismic to co-seismic) are measured by the red component, whereas the green component measures the backward (from co-seismic to pre-seismic) changes, and the blue means relatively no changes (between pre-seismic and co-seismic). As the buildings can maintain high coherence over the time, the decrease in co-seismic coherence from the pre-seismic coherence is used as a proxy of the earthquake induced damage. In this research, a new component called the backward change is added to visualize the retrospective changes caused by factors other than the earthquake. Higher average values of the pre-seismic and co-seismic coherences accounted for by the third change-free parameter implies lower changes in both directions (backward or forward).

**Figure 5:**
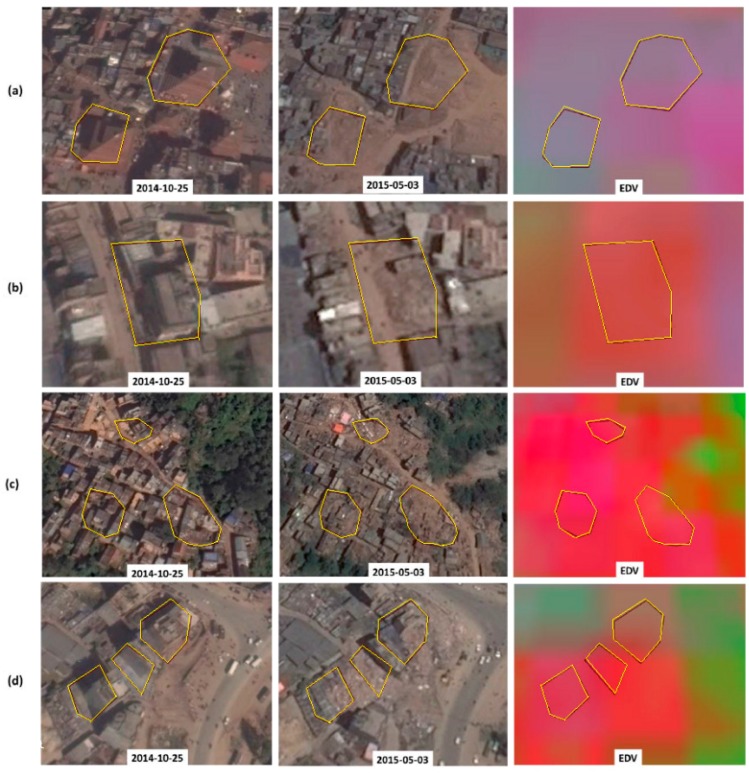
Performance of the EDV in different locations (**a**–**d**) that were highly damaged by the earthquake. The blocks of highly damaged (lethal) buildings are delineated by yellow polygons in each image. The left and middle columns show pre-seismic and post-seismic Google Earth images, whereas the right column shows the EDV image. The date of the Google Earth image is labeled in each image. The amount of redness in the EDV indicates severity of the building damage.

**Figure 6:**
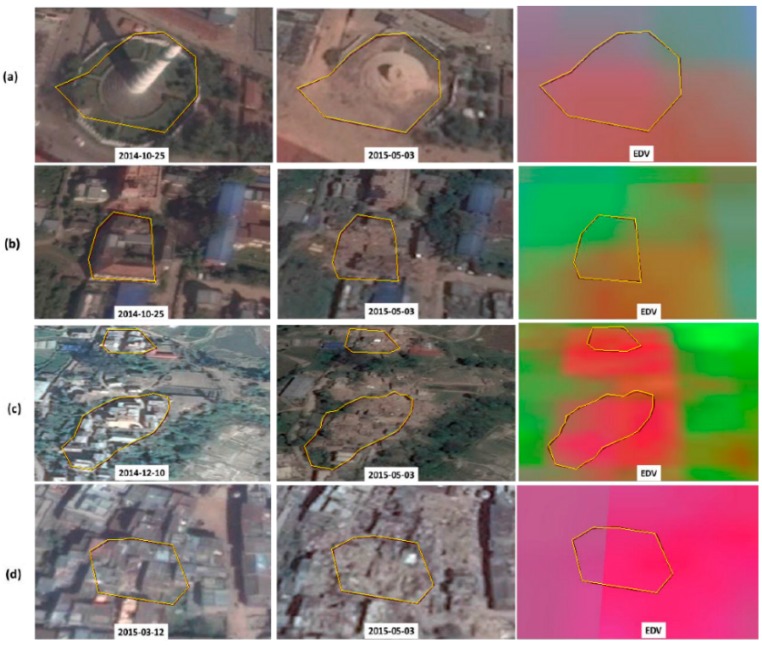
Performance of the EDV in different locations (**a**–**d**) that were highly damaged by the earthquake. The blocks of highly damaged (lethal) buildings are delineated by yellow polygons in each image. The left and middle columns show pre-seismic and post-seismic Google Earth images, whereas the right column shows the EDV image. The date of the Google Earth image is labeled in each image. The amount of redness in the EDV indicates severity of the building damage.

The performance of the EDV is demonstrated in a number of highly damaged (lethal) locations (Figure 5 and Figure 6). The redness of the EDV imagery indicates severity of the building damage. As most of the lethal polygons have a higher amount of redness (than greenness or blueness), the EDV has visualized the building damage efficiently. Nevertheless, in some locations (Figure 5a and Figure 6b), EDV is dim to visualize the damage.

It should be noted that the red pixels of the EDV imagery (Figure 4) are not always matched with the highly damaged areas. Similar to the earthquake induced building damage, growth of trees can also drop the coherence, and it can be confused with building damage. Therefore, detection of building damage seems more difficult if buildings are inter-mixed with trees.

### 3.2. Comparison to the NDPM

**Figure 7:**
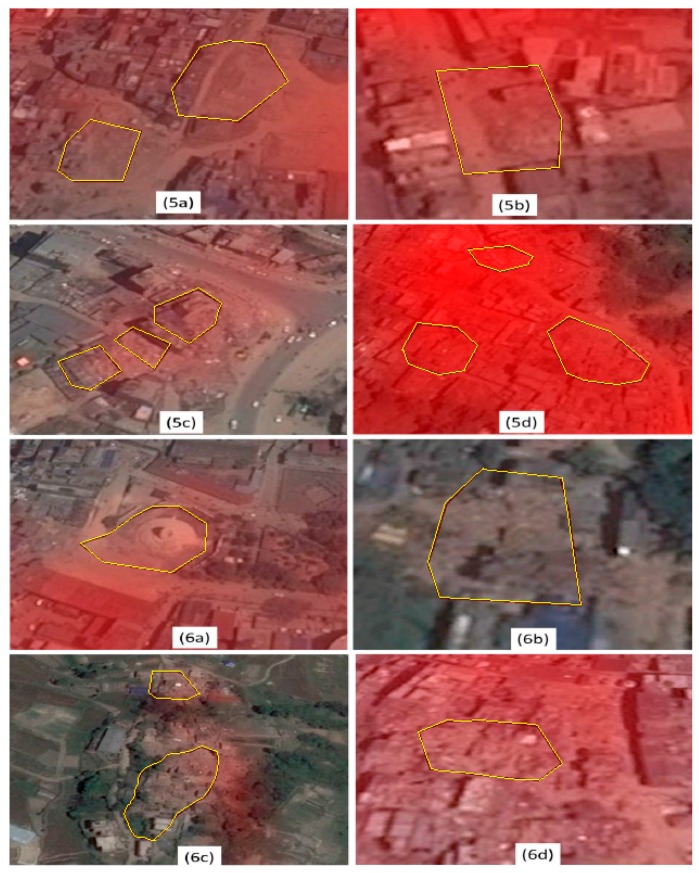
The NASA Damage Proxy Map (NDPM) in a number of highly damaged locations overlaid on the Google Earth imagery dated 3 May 2015 (post-seismic). The labels 5a to 5d and 6a to 6d in this figure denote the corresponding locations described in Figure 5 and Figure 6, respectively. The amount of redness in the NDPM imagery indicates severity of building damage.

The performance of the NDPM in a number of locations is demonstrated in Figure 7. The amount of redness reflects the severity of building damage. However, many highly damaged locations are lacking redness completely or partially. While comparing the Figure 6b with the label 6b of the Figure 7, NDPM is missing the lethal blocks completely. Contrary to the EDV, the NDPM is very dim for all highly damaged locations except for the location 5d. As the same scale of the redness is used, it is difficult to distinguish the damaged buildings using the NDPM. However, the EDV has demonstrated high contrast in most of the locations (Figure 5 and Figure 6), and it is more sensitive to the damaged buildings than the NDPM.

### 3.3. Cross-Validation Results

Following the visual performance in previous sections, the performance of the EDV was compared to the NDPM quantitatively by using all ground truth polygons (90 for each class) prepared. The overall accuracy (kappa coefficient) of EDV and NDPM calculated from 10-fold cross-validation method using four supervised classifiers (k-Nearest Neighbors, Gaussian Naïve Bayes, Random Forests, and Support Vector Machine) are shown in Table 1.

Among the supervised classifiers tested, the EDV performed better than the NDPM in terms of overall accuracy and kappa coefficient. Out of four classifiers, Gaussian Naïve Bayes showed the best performance for both the EDV (overall accuracy = 0.86, Kappa coefficient = 0.71) and NDPM (overall accuracy = 0.71, Kappa coefficient = 0.41) followed by the k-Nearest Neighbors and Random Forests.

There is a significant difference between the performance of EDV and NDPM; the highest kappa coefficient achieved with the Gaussian Naïve Bayes is 0.41 in the case of NDPM, whereas the EDV achieved 0.71 kappa coefficient using the same classifier. The NDPM used only the HH polarization (single band information) based pre-seismic and co-seismic coherence change method. On the contrary, the EDV deployed both the HH and HV polarizations; normalized differences of the pre-seismic and co-seismic coherences, and vice versa, as well as the third change-free parameter. The accuracy metrics, overall accuracy (kappa coefficient) based on different supervised classifiers: k-Nearest Neighbors, Gaussian Naïve Bayes, Random Forests, and Support Vector Machine were 0.69 (0.39), 0.77 (0.53), 0.61 (0.22), and 0.71 (0.41), respectively, using a single HH polarization based forward change parameter as in the case of NDPM. The overall accuracy (Kappa coefficient) obtained from a single HH polarization based forward change parameter is significantly lower than the overall accuracy (Kappa coefficient) obtained from the EDV. The EDV provides improved detection of the building damage by adding additional polarization and more change parameters over the NDPM.

## 4. Conclusions

An improved technique, the EDV was proposed in the research for visualizing the earthquake-induced building damage. The EDV consists of the forward (from pre-seismic to co-seismic), backward (from co-seismic to pre-seismic), and change-free (between pre-seismic and co-seismic) parameters. The performance of the EDV was assessed in the Kathmandu Valley, which was severely shaken by the 2015 Nepal Earthquake. The cross-validation results showed that the EDV is more sensitive to the building damage than the existing NDPM method. The improvement was achieved by using an additional polarization (HV) and more change parameters. False detection of the buildings damage in forested areas due to growth of trees over the time was observed. Forested areas could be masked out in advance or the false detection could be minimized if the short time span images are available.

The earthquake induced hazards such as ground shaking/displacement, soil liquefaction, flooding/tsunami, and fires can damage buildings and man-made structures. The major cause of casualties of the earthquake is the damage of buildings and man-made structures where most of the human activities occur. Therefore, detection of building/structure damage is important for identifying the location of trapped victims during the immediate post-disaster period, and assisting the deployment of emergency services in the trapped houses. Though EDV provides better and rapid damage information based on coherence change measures using multiple SAR polarizations, tracking the individual damaged buildings is very challenging. Availability of sub-meter resolution SAR data is expected in the future to overcome the challenge. However, the availability of near-real time SAR data cannot be assured. For instance, a post-earthquake ALOS-2 SAR image was captured on 2 May, which is seven days after the strike of the 2015 Nepal Earthquake. This limits the usefulness of the satellite data in terms of detecting damage and saving life, as the best time has already passed. Moreover, planning the orbit of satellite to capture earthquake damage is difficult due to the uncertain nature of the earthquakes. Near real-time observations of the earthquake-prone fault zones using geostationary satellites or satellites with rapid revisits are suggested. Airborne lidar imaging can also be a promising technique in this kind of situation. Nevertheless, development of local telecommunication technology is inevitable for saving lives during the disaster. On the other hand, better building damage information obtained from the EDV is expected to provide new insights into disaster-preparedness planning such as updating building codes in the vulnerable fault zones, and allocating open spaces and evacuation zones.

## Figures and Tables

**Table 1:** Performance of Earthquake Damage Visualization (EDV) and NASA Damage Proxy Map (NDPM) based on statistical metrics, overall accuracy (kappa coefficient) calculated from 10-fold cross-validation method using four supervised classifiers.

Methods	k-Nearest Neighbors	Gaussian Naïve Bayes	Random Forests	Support Vector Machine
EDV	0.82 (0.64)	0.86 (0.71)	0.76 (0.52)	0.69 (0.38)
NDPM	0.60 (0.20)	0.71 (0.41)	0.61 (0.21)	0.59 (0.17)
